# Relationship between serum total bilirubin and frailty in middle-aged and elderly individuals

**DOI:** 10.3389/fmed.2025.1567050

**Published:** 2025-07-08

**Authors:** Cunbao Ling, Libing Tian, Chunyan Zhang, Yaping Tian

**Affiliations:** ^1^Department of Medical Innovation Research, Chinese PLA General Hospital, Beijing, China; ^2^School of Basic Medical Sciences, Jiangsu Medical College, Yancheng, Jiangsu, China; ^3^State Key Laboratory of Kidney Diseases, Chinese PLA General Hospital, Beijing, China

**Keywords:** bilirubin, frailty, middle-aged and older adults, NHANES, cross-sectional study

## Abstract

**Background:**

In recent years, the increasing number of elderly individuals has highlighted frailty as a significant public health issue. Although the potential health benefits of bilirubin in adults are recognized, studies on the link between bilirubin levels and frailty are sparse. This study explores the association between serum total bilirubin (STB) and frailty in individuals aged 45–85 who participated in the National Health and Nutrition Examination Survey (NHANES) from 2015 to 2020.

**Materials and methods:**

STB levels were measured using the Jendrassik-Grof method. Frailty was evaluated using a frailty index that included 49 deficits across seven domains. Survey weighted logistic regression analyses and Restricted Cubic Spline (RCS) techniques were used to examine the relationship between STB and frailty. Subgroup analyses were performed to confirm the consistency of the observed association.

**Results:**

The study comprised a cohort of 8,603 individuals aged between 45 and 85 years, of whom 54.7% were female, and 3,037 were identified as frail. In models that were fully adjusted, each unit increase in STB was associated with a 5% reduction in the risk of frailty. Participants in the second and third tertiles of STB exhibited statistically significant lower odds of frailty compared to those in the lowest tertile, with odds ratios (ORs) of 0.75 and 0.59, respectively. RCS analysis revealed an L-shaped correlation between STB levels and frailty, exhibiting statistically significant non-linearity (*P* = 0.0075), with an inflection point at 17.1 μmol/L of STB (the upper limit of normal). Below this threshold, a negative correlation is evident, whereas a weak positive correlation is observed for values exceeding 17.1 μmol/L. Further subgroup analysis within the physiological range of bilirubin suggests that the negative association between STB and frailty is more pronounced in individuals younger than 60 years.

**Conclusion:**

This study reveals a negative relationship between STB levels and frailty among middle-aged and elderly individuals, suggesting that elevated bilirubin concentrations within the physiological range may reduce the risk of frailty.

## Background

Frailty, characterized by a decline across various physiological systems, is highly vulnerable to both internal and external stressors. This vulnerability markedly increases the risk of numerous adverse health events such as falls, disabilities, hospitalizations, and impaired quality of life, ultimately increasing the risk of mortality (1, 2). As the population ages, the prevalence of frailty is rising, imposing a considerable burden on individuals, families, and the public healthcare system (1). Frailty assessment is conducted using a frailty index, which evaluates multidimensional health deficits (3). Previous research has suggested that biological mechanisms underlying frailty may include oxidative stress, inflammatory responses, insulin resistance, and metabolic syndrome (4–6). Further investigation is needed to enhance the prevention and treatment of frailty by identifying new risk factors.

Bilirubin, a pigment with a tetrapyrrole ring structure, is the end-product of heme catabolism in the bloodstream (7). Studies have revealed that bilirubin exhibits significant antioxidant properties due to the active hydrogen atom located at the C-10 bridge position of its tetrapyrrole ring (8). It surpasses vitamin E in its ability to clear lipid peroxides. Beyond its antioxidant capabilities, bilirubin also exerts anti-inflammatory effects (9). These properties help reduce the risk of several chronic conditions, including cardiovascular diseases, metabolic syndrome, and chronic kidney disease (10, 11). Elite athletes demonstrate notably increased serum bilirubin concentrations, with a significantly higher prevalence of Gilbert’s syndrome (a form of congenital unconjugated hyperbilirubinemia) observed in this group compared to the general population (12). These findings imply that mildly elevated serum bilirubin levels may play a role in enhancing athletic performance (13). Despite extensive research into bilirubin’s biological effects, studies on the correlation between bilirubin and frailty in large populations remain limited. Thus, this study seeks to explore the relationship between bilirubin and frailty to provide new insights into the prevention and management of frailty.

## Materials and methods

### Data source and subjects

**FIGURE 1:**
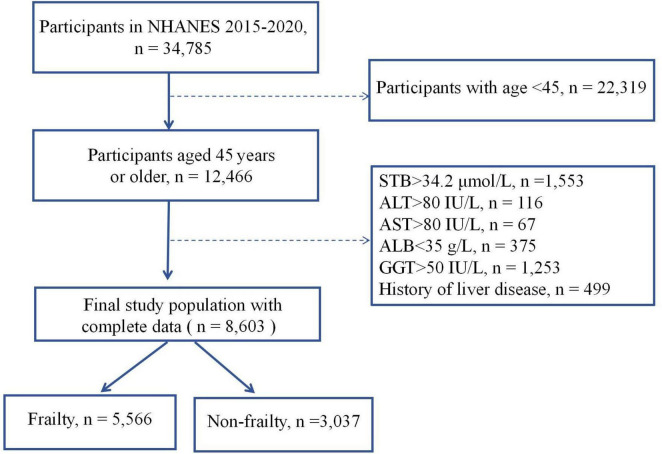
Flowchart of sample inclusion and exclusion. Dotted lines indicate the exclusion process.

NHANES, a comprehensive epidemiological survey conducted by the National Center for Health Statistics (NCHS), focuses on the health and nutrition of the non-institutionalized civilian population of the US. The NHANES protocols received approval from the ethics review board of the NCHS, with all participants providing informed consent. Further details about NHANES can be found on the official website.^1^ Our analysis included all subjects from the 2015 to 2020 datasets aged 45–85 years who had complete data for frailty and STB (*n* = 12,466). To minimize the confounding influences on serum bilirubin concentrations, individuals with existing liver conditions or liver impairment were not included in the study. The exclusion criteria included serum total bilirubin levels greater than 34.2 μmol/L, albumin concentration < 35 g/L, aspartate aminotransferase (AST) levels exceeding 80 IU/L, alanine aminotransferase (ALT) levels above 80 IU/L, gamma-glutamyl transferase (GGT) levels surpassing 50 IU/L, or a self-reported history of liver disease. The determination of liver disease was based on participants’ affirmative answers to the question, “Has a doctor or other health professional ever informed you of any liver condition?.” Additionally, participants lacking data for covariates were excluded. Ultimately, the study comprised 8,603 participants, as shown in Figure 1.

### Variables

Frailty was evaluated using the frailty index developed by Hakeem et al. (14). This index includes 49 deficits across seven main domains: Cognitive function (1 item), dependence (15 items), depressive symptoms (7 items from the Patient Health Questionnaire PHQ-9), comorbidities (13 items), hospital utilization and general health (5 items), physical performance and anthropometry (2 items, including handgrip strength and body mass index), and critical lab values (6 items). Details are provided in Supplementary Table 1. Deficits were scored on a scale from 0 (no deficit) to 1 (severe deficit), enabling the integration of continuous and categorical data. The overall frailty index score was calculated as the proportion of observed deficits to the total, with a cutoff value of 0.21 to distinguish frail (> 0.21) from non-frail (≤ 0.21) individuals (14, 15).

With the Beckman DXC800 (Beckman, United States), STB was measured using the timed-endpoint Diazo method (Jendrassik-Grof), in which bilirubin converts to azobilirubin by reacting with caffeine, benzoate, and acetate. During a specified time, the absorbance at 520 nm is monitored to quantify STB.

A comprehensive evaluation of potential covariates was considered, including age, gender, race, marital status, family income, education level, smoking, alcohol consumption, ALT, GGT, Diabetes mellitus (DM), and hypertension. Depending on marital status, respondents were categorized as married, widowed, divorced, separated, or never married. The individual’s educational attainment was divided into three categories: Less than high school, high school, and beyond high school. PIR was dichotomized into below-poverty (< 1) and above-poverty (≥ 1). In terms of alcohol consumption, there were three categories: Never (zero drinks in a lifetime), current (12 drinks and currently drinking), and former (zero drinks last year but 12 drinks in a lifetime). The behavior of smoking was classified into three categories: Non-smokers, former smokers, and current smokers. Normal weight is defined as a BMI of 18.5–24.9 kg/m^2^, overweight as a BMI of 25.0–29.9 kg/m^2^, and obesity as a BMI of ≥30 kg/m^2^. DM was categorized as yes or no, based on physician diagnosis, fasting blood glucose (≥ 7.0 mmol/L), glycated hemoglobin (HbA1c ≥ 6.5%), random blood glucose (≥ 11.1 mmol/L), or hypoglycemic drug application. Hypertension was also categorized as yes or no, based on physician diagnosis, systolic blood pressure (≥ 140 mmHg), diastolic blood pressure (≥ 90 mmHg), or the use of antihypertensive medications. This study adopts the Random Forest (RF) algorithm, a machine learning strategy, to fill in all missing values of covariates.

### Statistical analysis

To address the complex multistage sampling design of NHANES, appropriate sample weights were applied to enhance data precision following NHANES guidelines. Variables were presented as weighted mean ± standard error (SE) for continuous variables and “number, percentage” (n,%) for categorical variables. Weighted *t*-tests and weighted chi-squared tests were employed to assess the baseline characteristics of participants by frailty status for continuous and categorical variables, respectively. The relationship between STB and frailty was analyzed through both continuous and categorical approaches using survey weight-adjusted logistic regression models. The study incorporated three models: Unadjusted model; Model 1, adjusting for age; and Model 2, which additionally considered adjustments for gender, race, marital status, educational level, and family income, smoking status, alcohol consumption, BMI, ALT, GGT, DM, and hypertension.

Furthermore, a weighted Restricted Cubic Spline (RCS) was utilized to elucidate the dose-response relationship between STB concentration and frailty risk. Additionally, a subgroup analysis, stratified by variables such as age, gender, marital status, educational level, family income, smoking status, alcohol consumption, BMI, DM, and hypertension, was conducted to explore the potential modifying effects of covariates on the relationship between STB and frailty. In all statistical analyses, a *p*-value of less than 0.05 was considered statistically significant on a two-sided test using R software (version 4.2.2) (16).

## Results

### Participants’ baseline characteristics

The study included 8,603 participants, comprising 4,098 males and 4,505 females (Table 1). The prevalence of frailty was 35.3% (*n* = 3,037), with statistically significant differences noted across all variables. Frail participants were older on average (65.31 ± 0.29 years) compared to non-frail participants (60.05 ± 0.27 years). A lower level of STB was observed in the frail group (7.6 ± 0.10 μmol/L) vs. the non-frail group (8.82 ± 0.09 μmol/L). Furthermore, frailty was more prevalent among females, individuals living alone (including those who were widowed, divorced, separated, or never married), persons with lower family incomes, those with less than a high school education, and individuals who were current or former smokers, former alcohol consumers, obese, or diagnosed with DM or hypertension.

**TABLE 1:** Weighted baseline characteristics of participants with and without frailty in the enrolled population of NHANES.

Variables	Total	Non-frailty	Frailty	*P-*value
	*N* = 8,603	*N* = 5,566	*N* = 3,037	
Age (years), Mean ± S.E	61.47 ± 0.23	60.05 ± 0.27	65.31 ± 0.29	<0.0001
Age groups				<0.0001
≤ 60 years	3,752 (50.36)	2,779 (55.77)	973 (35.73)	
> 60 years	4,851 (49.64)	2,787 (44.23)	2,064 (64.27)	
STB (μmol/L), Mean ± S.E	8.49 ± 0.09	8.82 ± 0.11	7.60 ± 0.10	<0.0001
STB groups				<0.0001
≤ 17.1 μmol/L	8,311 (95.90)	5,355 (95.12)	2,956 (98.02)	
> 17.1 μmol/L	292 (4.10)	211 (4.88)	81 (1.98)	
Gender, *n* (%)				<0.0001
Male	4,098 (45.30)	2,750 (47.62)	1,348 (39.00)	
Female	4,505 (54.70)	2,816 (52.38)	1,689 (61.00)	
Race, *n* (%)				<0.0001
Mexican American	1,033 (5.62)	714 (5.70)	319 (5.43)	
Non-Hispanic Black	2,008 (9.49)	1,163 (7.82)	845 (14.00)	
Non-Hispanic White	3,335 (71.19)	2,121 (73.00)	1,214 (66.29)	
Other	2,227 (13.70)	1,568 (13.48)	659 (14.28)	
Marital status, *n* (%)				<0.0001
Married/living with partner	5,191 (66.97)	3,648 (71.10)	1,543 (55.78)	
Widowed/divorced/separated	2,745 (27.14)	1,541 (23.60)	1,204 (36.74)	
Never married	667 (5.89)	377 (5.30)	290 (7.48)	
Family income, *n* (%)				<0.0001
Above poverty level	7,360 (91.71)	4,927 (94.11)	2,433 (85.21)	
Below poverty level	1,243 (8.29)	639 (5.89)	604 (14.79)	
Education level, *n* (%)				<0.0001
Less than high school	1,888 (11.72)	1,050 (9.31)	838 (18.25)	
High school	2,040 (24.58)	1,234 (22.73)	806 (29.58)	
Beyond high school	4,675 (63.70)	3,282 (67.96)	1,393 (52.17)	
Smoking, *n* (%)				<0.0001
Never	4,795 (55.80)	3,381 (59.65)	1,414 (45.36)	
Former	2,528 (30.67)	1,460 (28.68)	1,068 (36.04)	
Current	1,280 (13.54)	725 (11.67)	555 (18.59)	
Alcohol consumption, *n* (%)				<0.0001
Never	1,093 (9.31)	771 (9.60)	322 (8.50)	
Former	1,070 (9.75)	562 (7.39)	508 (16.13)	
Current	6,440 (80.94)	4,233 (83.00)	2,207 (75.36)	
BMI, *n* (%)				<0.0001
Normal	1,993 (23.43)	1,492 (26.24)	501 (15.83)	
Overweight	3,038 (34.85)	2,061 (36.35)	977 (30.79)	
Obesity	3,572 (41.72)	2,013 (37.41)	1,559 (53.38)	
DM, *n* (%)				<0.0001
No	6,212 (78.18)	4,544 (85.19)	1,668 (59.20)	
Yes	2,391 (21.82)	1,022 (14.81)	1,369 (40.80)	
Hypertension, *n* (%)				<0.0001
No	3,759 (49.28)	3,084 (58.67)	675 (23.86)	
Yes	4,844 (50.72)	2,482 (41.33)	2,362 (76.14)	

Percentage for categorical variables, mean ± SE for continuous variables. SE, standard error; STB, serum total bilirubin; BMI, body mass index, DM, Diabetes mellitus.

### The relationship between STB and frailty

The relationship between STB levels and frailty was examined utilizing weighted logistic regression analysis, as presented in Table 2. In both the unadjusted model and Model 1(adjusting for age), an increase of one μmol/L in STB was associated with a 7% decrease in the likelihood of frailty [odds ratio (OR) = 0.93; *P* < 0.0001]. In Model 2, which accounted for all potential confounding variables, the reduction in frailty risk persisted at 5% per 1 μmol/L increase in STB (OR = 0.95; *P* < 0.0001). To further investigate this association, STB levels were divided into tertiles, with the lowest tertile serving as the reference category. In Model 1, the OR for the second tertile (T2) relative to the lowest tertile (T1) was 0.69 (P for trend < 0.0001). In Model 2, the OR for T2 remained statistically significant (OR = 0.75; P for trend < 0.0001). Comparable findings were observed for the third tertile (T3), with an OR of 0.46 (P for trend < 0.0001) in Model 1 and an OR of 0.59 (P for trend < 0.0001) in Model 2.

**TABLE 2:** Association between STB and frailty.

	Unadjusted model		Model 1		Model 2	
	ORs (95%CI)	*P*-value	ORs (95%CI)	*P*-value	ORs (95%CI)	*P-*value
STB (continuous)	0.93 (0.92,0.95)	< 0.0001	0.93 (0.91,0.94)	< 0.0001	0.95 (0.93,0.97)	< 0.0001
**STB (tertiles)**						
T1 (*N* = 2,936)	1(Ref)		1(Ref)		1(Ref)	
T2 (*N* = 3,031)	0.72 (0.61,0.85)	<0.001	0.69 (0.58,0.83)	<0.001	0.75 (0.61,0.92)	0.01
T3 (*N* = 2,636)	0.50 (0.42,0.59)	<0.0001	0.46 (0.38,0.55)	< 0.0001	0.59 (0.50,0.70)	<0.0001
*P* for Trend		<0.0001		<0.0001		<0.0001

Tertile 1 of STB (T1, ≤ 5.13 μmol/L), Tertile 2 of STB (T2, 5.14–8.55 μmol/L), Tertile 3 of STB (T3, 8.56–34.2 μmol/L). No covariates were adjusted in the Crude model. Model 1 was adjusted for age, while Model 2 was additionally adjusted for gender, race, marital status, educational level, and family income, smoking status, alcohol consumption, body mass index, ALT, GGT, Diabetes mellitus and hypertension. OR, odds ratio; CI, confidence interval, ref, reference; STB, serum total bilirubin; T, tertile.

**FIGURE 2:**
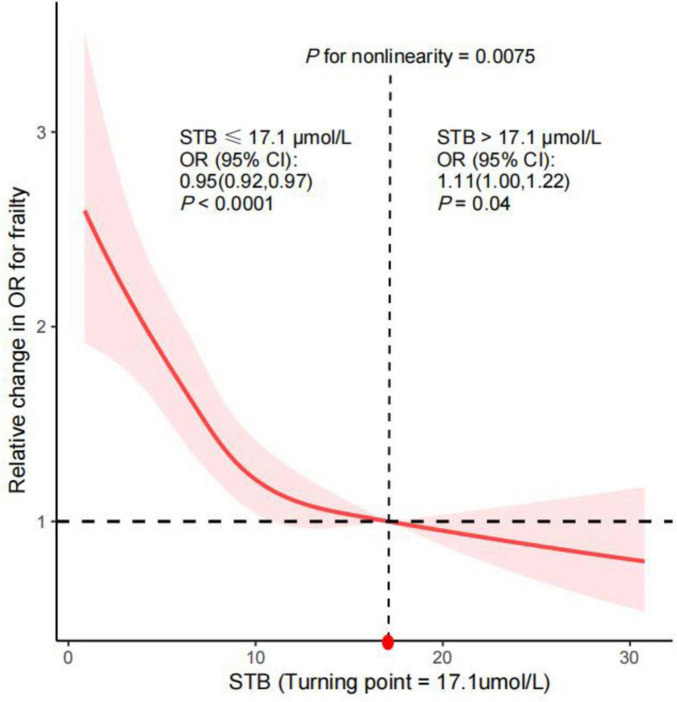
RCS analysis with multivariate-adjusted associations between frailty and STB. The model was adjusted for age, gender, race, marital status, educational level, family income, smoking status, alcohol consumption, and body mass index, ALT, GGT, Diabetes mellitus and hypertension.

The RCS analyses were performed to address potential bias from assumed linearity and to assess the dose-response relationships between predictors and outcomes accurately. It was determined that STB levels and frailty risk exhibit an L-shaped relationship after adjusting for potential confounders, as indicated by a non-linear *P*-value of less than 0.0075 (refer to Figure 2). The threshold point was identified as 17.1 μmol/L, the upper normal limit of STB. For STB levels less than or equal to 17.1 μmol/L, a negative association with frailty was observed, with an OR of 0.95 and a *P*-value of less than 0.0001. Conversely, for STB levels greater than 17.1 μmol/L, a positive association with frailty was observed, with an OR of 1.11 and a *P*-value of 0.04.

### Stratified analyses and interaction test

**FIGURE 3:**
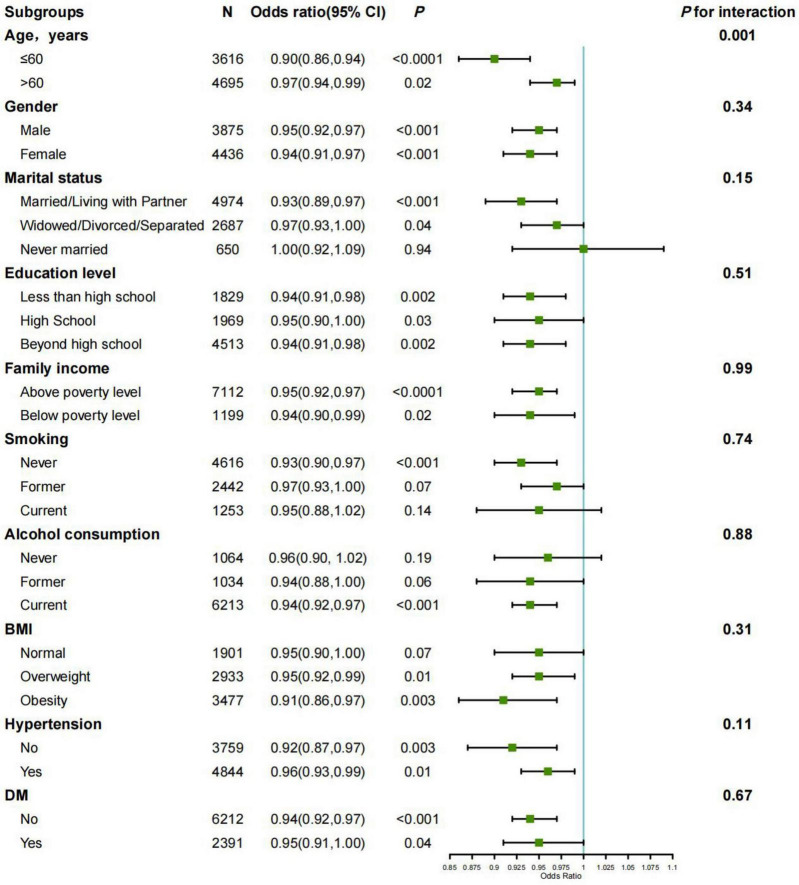
Associations between STB (≤ 17.1 μmol/L) and frailty in subgroups. Estimates were adjusted for age, gender, race, marital status, educational level, family income, smoking status, alcohol consumption, BMI, ALT, GGT, DM, and hypertension. The stratification variable was not included in the adjusted model in each subgroup analysis in every case. OR, odds ratio; CI: confidence interval; BMI: body mass index; DM, Diabetes mellitus.

To evaluate the robustness of the observed negative associations, subgroup analyses of logistic regression were performed utilizing Model 2, as illustrated in the forest plot in Figure 3. The findings of our study demonstrated no significant interaction effects influencing the relationship between STB and the incidence of frailty when stratified by variables such as gender, marital status, educational level, family income, smoking status, alcohol consumption, BMI, hypertension, or DM, with *P* for interaction ranging from 0.11 to 0.99. Notably, age stratification revealed a significant interaction effect (*P* for interaction = 0.001). The negative association between frailty and STB was more pronounced in the subgroup aged under 60 years (OR = 0.9, *P* < 0.0001).

## Discussion

To the best of our knowledge, this research is the first comprehensive analysis to investigate the correlation between STB levels and frailty, using large-scale, representative population data from NHANES. After controlling for various confounding variables, our study identified an L-shaped correlation between STB levels and frailty. Notably, a significant inverse dose-response relationship was observed at STB concentrations of ≤ 17.1 μmol/L, indicating that elevated bilirubin levels within the physiological range may serve as a protective factor against the development of frailty, particularly in individuals under the age of 61 years.

Frailty is characterized by a decline in physiological functions and increased vulnerability to minor stressors, which elevates the risk of chronic diseases, disability, and mortality. Increased oxidative stress and persistent inflammatory states are significant pathological contributors to frailty (17–19). Previous research has shown that individuals with frailty exhibit elevated levels of inflammatory markers such as C-reactive protein (CRP), interleukin-1 beta (IL-1β), and IL-6, accompanied by chronic systemic inflammation and increased monocyte counts (5, 20). Additionally, frail individuals experience heightened oxidative stress, which contributes to age-related muscle fiber atrophy through oxidative damage-induced apoptosis (21, 22). Furthermore, frailty is associated with insulin resistance, disrupting the body’s balance between oxidation and antioxidation, ultimately aggravating inflammatory responses, particularly affecting adipocytes and macrophages in adipose tissue while also reducing muscle mass density (19).

A reduction in the capacity to execute activities of daily living (ADLs) serves as a defining characteristic of frailty (23). Within this framework, our results mirror those of Inoguchi et al., who analyzed 198 Japanese diabetic patients aged 70 years and above, showing that decreased serum bilirubin levels are significant predictors of impaired ADL performance. Nonetheless, our research includes a wider range of adults between 45 and 85 years, improving the representativeness and applicability of our results.

Bilirubin is noted for its strong antioxidant capabilities and its linkage to a lower incidence of several chronic conditions, such as cardiovascular diseases, metabolic syndrome, insulin resistance, diabetes, atherosclerosis, and chronic kidney disease (24–29). The alleviation of these disorders is primarily ascribed to the antioxidant and anti-inflammatory properties of bilirubin (30, 31). Even at minimal concentrations of 10 nM, bilirubin is capable of safeguarding neurons exposed to hydrogen peroxide (H_2_O_2_) at levels 10,000 times the physiological norm, shielding them from oxidative stress-induced harm (32). Moreover, bilirubin diminishes the expression of adhesion molecules, suppresses T-cell activation, and reduces the levels of pro-inflammatory cytokines (30).

In our study, we noted that bilirubin’s role in protecting against frailty presents a threshold effect. This observation is consistent with prior studies by Tang et al., which revealed a negative correlation between STB levels and the risk of hypertension at concentrations up to 12.17 μmol/L, confirming that higher bilirubin levels within physiological limits offer health advantages (33). Conversely, excessively elevated serum bilirubin levels might intensify oxidative stress and inflammation. We propose that bilirubin concentrations not surpassing 17.1 μmol/L could prevent frailty through its antioxidant and anti-inflammatory mechanisms. This proposition requires further empirical examination and corroboration in subsequent studies.

The principal advantage of this investigation stems from its reliance on nationally representative multi-ethnic survey data, which enables precise stratification of analyses by numerous variables while accounting for a variety of confounders. Our findings indicated that the negative correlation between STB and the incidence of frailty remained consistent across a range of demographic and lifestyle characteristics, including gender, marital status, educational attainment, family income, smoking status, alcohol consumption, BMI, DM, and hypertension, with no significant interactions observed. It is important to acknowledge certain limitations, with the cross-sectional nature of this study being a significant constraint. It hinders the establishment of a causal link between STB levels and frailty. Secondly, although adjustments were made for numerous confounding variables, the potential presence of unaccounted confounders might skew the results. Lastly, the absence of differentiation between conjugated and unconjugated bilirubin in our analysis curtails our capacity to discern which form is more significantly linked to frailty. Future investigations should elucidate the specific mechanisms involved, and we advocate for the execution of prospective large-scale studies to substantiate the predictive capacity of serum bilirubin levels concerning frailty.

## Conclusion

This research confirmed that higher STB levels, within the physiological range, are negatively related to frailty among adults aged 45–85 years old. Should future studies verify a causal connection, bilirubin could potentially be targeted as a therapeutic measure to reduce frailty risk.

## Data Availability

Publicly available datasets were analyzed in this study. This data can be found at: The dataset originates from the National Health and Nutrition Examination Survey (https://www.cdc.gov/nchs/nhanes/), which is accessible online via the Centers for Disease Control and Prevention (CDC).

## Footnotes

1
https://www.cdc.gov/nchs/nhanes/
